# Music Listening Behavior, Health, Hearing and Otoacoustic Emission Levels 

**DOI:** 10.3390/ijerph110807592

**Published:** 2014-07-25

**Authors:** Kathleen Hutchinson Marron, Brittany Sproat, Danielle Ross, Sarah Wagner, Helaine Alessio

**Affiliations:** 1 Department of Speech Pathology and Audiology, Miami University, Oxford, OH 45056, USA; 2 MJ Care, Milwaukee, WI 53227, USA; E-Mail: Sproatba@miamioh.edu; 3 Department of Kinesiology and Health, Miami University, Oxford, OH 45056, USA; E-Mails: rossdk@miamioh.edu (D.R.); alessih@miamioh.edu (H.A.); 4 Helping Hands Center for Special Needs, Columbus, OH 45056, USA; E-Mail: swags89@gmail.com

**Keywords:** hearing level, otoacoustic emission, cardiovascular health, waist-to-hip ratio, body mass index

## Abstract

This study examined the relationship between hearing levels, otoacoustic emission levels and listening habits related to the use of personal listening devices (PLDs) in adults with varying health-related fitness. Duration of PLD use was estimated and volume level was directly measured. Biomarkers of health-related fitness were co-factored into the analyses. 115 subjects ages 18–84 participated in this study. Subjects were divided into two sub-groups; PLD users and non-PLD users. Both groups completed audiological and health-related fitness tests. Due to the mismatch in the mean age of the PLD user *versus* the non-PLD user groups, age-adjusted statistics were performed to determine factors that contributed to hearing levels. Age was the most significant predictor of hearing levels across listening and health-related fitness variables. PLD user status did not impact hearing measures, yet PLD users who listened less than 8 hours per week with intensities of less than 80 dBA were found to have better hearing. Other variables found to be associated with hearing levels included: years listening to PLD, number of noise environments and use of ear protection. Finally, a healthy waist-to-hip ratio was a significant predictor of better hearing, while body mass index approached, but did not reach statistical significance.

## 1. Introduction

Hearing loss is the third most prevalent chronic health condition within the adult population in the United States [1]. In the past 30 years, the prevalence of hearing loss has almost doubled and is expected to double again by 2050 [1]. Although the public generally attributes sensorineural hearing loss to presbycusis, hearing loss is becoming a growing concern for younger generations. This is due partly to noise exposure during recreational activities, including listening to music on Personal Listening Devices (PLD) [2]. The ability of PLDs to contribute to permanent hearing damage is particularly alarming because adolescent and young adult PLD users tend to be unaware that Noise Induced Hearing Loss (NIHL) can result from excessive PLD use [2]. 

NIHL is a preventable sensorineural hearing loss that begins with a high-frequency deficit and occurs gradually as the result of chronic noise exposure [3]. The increasing prevalence of NIHL can be attributed to several factors including occupational and leisure-noise exposure. Sources of leisure noise exposure include concerts, fireworks, construction sites, firearms, and the subway [3]. Another source of leisure-noise that has become particularly popular in the last 20 years is the PLD [4]. Excessive use of PLDs with earbuds has the capability of causing both temporary and permanent hearing loss by inflicting damage to the cochlea [5]. Although The Occupational Safety and Health Administration (OSHA) guidelines limit noise exposure in the occupational setting, few, if any, such restrictions apply to the non-occupational or leisure setting [6]. 

The impact of PLD use on hearing must be examined in the context of the intensity of sound as well as the duration of listening. While individual susceptibility exists, higher intensities and longer durations of noise exposure damage ears more than lower intensities and shorter durations [7]. Given the capability of PLDs to store excessive quantities of music, the long battery life and the ability of these devices to play music at hazardously high sound pressure levels, the possibility of increased hearing loss due to PLD use has become an ever-present concern [2,7,8]. A study by Meyer-Birsch [9] found that young adults who used their personal music system for more than seven hours a week had poorer hearing thresholds compared with other young adults who use PLDs less frequently. Personal listening devices also have the potential of producing output levels as high as 128 dB A-weighting filter (dBA), with typical PLD users listening at levels between 75 dBA and 110 dBA [7,10]. These typical listening levels may fall outside of the NIOSH guidelines of safe listening, as sounds exceeding 85 dBA for 8 hours or more are considered to be hazardous to an individual’s hearing [10]. 

Although the majority of PLD users listen within the safe listening range, 15–25% of PLD users exceed safe listening levels [11]. While preferred listening levels often fall within the acceptable range, listening levels may increase anywhere from 6 to 10 dBA when background noise is introduced in the listening environment [12]. This indicates that given the presence of background noise, PLD users may adjust their volume to unsafe listening levels. Furthermore, although the majority of listeners may be listening at a level or duration that is not sufficient to cause hearing loss, it is unlikely that music is PLD users only source of recreational noise exposure. The cumulative effects of recreational noise exposure may lead to an increase in the prevalence of hearing loss [13]. 

Moreover, NIHL is not the only cause of hearing loss. There are several uncontrollable factors leading to hearing loss, including genetics, age, gender, and race [10,14]. Many factors increase the risk for developing age-related hearing loss including non-modifiable factors such as cochlear aging and genetic predisposition (sex, race, specific genetic predisposition). Modifiable factors contributing to hearing loss may include environmental influences and health co-morbidities [15]. Despite efforts to improve modifiable behaviors linked to hearing acuity, physiological changes to parts of the ear including the inner and outer hair cells, the stria vascularis and afferent spiral ganglion neurons may lead to inevitable sensory, neuronal or metabolic age-related hearing loss [15].

Despite the fact that the prevalence of hearing loss is increasing, preventative measures such as noise exposure reduction, use of ear protection and increased health and fitness may have a positive impact on preserving hearing acuity, particularly with age [16]. In adult cohorts, high cardiovascular fitness, as measured with maximum oxygen consumption (VO_2_ max), has been shown to protect hearing, possibly due to increased internal auditory artery and cochlear blood flow [17]. This increased blood flow can in turn increase the availability of oxygen and nutrients to the structures of the inner ear, potentially rendering the ear less susceptible to the detrimental effects of noise and loud music [1]. The beneficial impact of high cardiovascular fitness on hearing acuity can be observed in terms of both preserved hearing acuity with age and resistance to Temporary Threshold Shift (TTS). 

Research by Hutchinson and Alessio has consistently demonstrated that individuals with high cardiovascular fitness, measured by VO_2_ max levels, have the best hearing sensitivity [16]. It is hypothesized that a healthy cardiovascular system attenuates the effects of age on hearing processes, thus inhibiting presbycusis and maintaining hearing sensitivity [16]. What’s more is that high levels of cardiovascular fitness are even more significant for older individuals. The same study by Alessio and Hutchinson found that after age 50, persons with moderate cardiovascular fitness displayed better hearing at most frequencies than persons of the same age with lower cardiovascular fitness levels [16]. Cardiovascular fitness also appears to provide protection against TTS caused by noise exposure [18]. A study conducted by Ismail *et al.* found that following an 8-month cardiovascular fitness training program, subjects suffered less (TTS) and recovered hearing faster than an untrained control group [19]. Furthermore, high-fit individuals also tended to exhibit less TTS after exercising during noise exposure than low-fit individuals [18]. 

The objectives of the current study were to (1) determine if PLD users have worse hearing than non-PLD users (2) to determine if PLD users who listen to music louder than 80 dBA free field equivalent levels have worse hearing than PLD users who listen to music at levels less than 80 dBA (3) to determine what additional factors such as audiological history, hearing protection use, exposure to noise and amount of time listening to PLDs influence hearing acuity and (4) to examine which health-related fitness variables have a positive influence on hearing acuity. 

## 2. Methods

### 2.1. Subjects

A total of 115 subjects, ages 18–84, were recruited from the Oxford, Ohio area via flyers posted at locations throughout Oxford. Participants were scheduled to complete the kinesiology and audiometric portions of the study via email. Interested participants received an email detailing expectations for each part of the study. Upon arrival to the first test session participants completed an informed consent form approved by the Miami University Institutional Review Board. 

### 2.2. Acoustic Testing

Participants were categorized into two groups, earbud PLD users and non-PLD users. PLD users were instructed to bring their most frequently used PLD and headphones with them to testing. Both PLD users and PLD non-users began by completing a hearing health questionnaire investigating hearing health as well as past and present noise exposure. After completing the questionnaire, participants underwent otoscopy to examine health of the outer ear and tympanic membrane. Participants with visible cerumen occlusions or external ear abnormalities were excluded from the study. Participants were then screened for middle ear disease using tympanometry. Those with peak pressure within +50 to –200 daPa and compliance greater than 0.3 mL were included. 

All audiometric testing and Otoacoustic Emission (OAE) testing occurred in double-walled sound-attenuating booths. After confirming the physical health of the ear, the Hughson-Westlake method was used to obtain pure tone thresholds on a Grason-Stadler 61 diagnostic audiometer (Grason-Stadler, Eden Prairie, MN, USA) at frequencies of 0.25 kHz, 0.5 kHz, 1 kHz, 2 kHz, 3 kHz, 4 kHz, 6 kHz and 8 kHz. Pulsed pure tones were delivered through Etymotic Research-5A insert earphones (Etymotic Research, Elk Grove Village, IL, USA). All audiometers and acoustic instruments met current American National Standards Institute (ANSI) standards [20]. 

Distortion Product Otoacoustic Emission (DPOAE) (2f1-f2) amplitudes were measured using the Biologic Systems Scout OAE software version 3.45 system with two intensity levels from 2 kHz to 8 kHz. Testing was completed at a f_1_/f_2_ ratio of 1.22 from 2 kHz to 8 kHz at a 10 dB level difference between the primary tones (L1 > L2). The present study evoked the DPOAE at two intensity levels to investigate the amplitude differences at 45/55 dB SPL (moderate level) and 55/65 dB SPL (high level). Research has shown that OAE testing may be a possible predictor of future NIHL and OAEs may decrease in amplitude prior to changes in audiometric thresholds [21,22]. Due to the possibility that changes in OAEs may be early indicators of hearing loss, 43 of the 115 participants completed OAE testing in addition to the other audiologic testing. These participants included random participants in the first half of the year, as well as all new participants tested in the second half of the year. OAE testing was completed on one ear for each subject. The test ear was selected on the basis of the results of the immittance testing including: presence of an acoustic reflex at 100 dB SPL, normal maximum peak compliance, and better pure tone thresholds. If both ears displayed equal selection criteria, then one ear was selected at random. Following completion of OAE testing, the non-PLD users completed final questionnaires regarding listening habits and potential risks of hearing loss, and then testing was completed. 

Actual measurements of each participant’s PLD intensity levels were assessed with Audioscan Verifit VF-1 equipment (Audioscan, Dorchester, Ontario, Canada) and a probe microphone inserted into the ear. Calibration of the instrument occurred Monday of each week. A reference microphone was placed over the participant’s ear with a probe microphone tube inserted into the ear canal. The probe tubing was inserted at 28 mm for females and 30 mm for males [23] using otoscopy. A baseline measure was taken by recording an on-ear-measure of ambient background levels to ensure accurate placement and function of the microphone. An earbud was then placed over the probe microphone on the test ear to secure the position of the tube, and the opposite earbud was also placed into the non-test ear. Each participant was instructed to select a song of his/her choice and set the volume at the most frequently used listening level. An on-ear measure of this volume level was taken to determine the SPL output at the participant’s tympanic membrane. After the selected song played for 2-minutes, a 15-second long term average speech spectrum (LTASS) run provided dB SPL data in 1/3 octave intervals from 0.25 to 6 kHz. The third on-ear measure was obtained when the participant listened to a song in the presence of background noise. A pink noise stimulus [24] was selected on the basis of similarity in frequency response and amplitude to stimuli recorded from a New York subway, used by Worthington *et al.* [8]. Pink noise was directed at the participant’s test ear at 75 dB SPL using a Hewlett Packard L2045W Computer (Hewlett Packard, Palo Alto, CA, USA) and Altec Lansing speaker model S90 (Altec Lansing, New York, NY, USA). Following initiation of background noise, participants were instructed to adjust their volume to meet their needs in the listening environment. The background noise was then removed, and the final volume measurement was completed. Graphic and tabular data were saved to a secure USB drive for later review and analysis. Two of the amplitude measurements made in quiet and in noise were converted to A-weighted free field equivalents for comparison to noise exposure standards. The Microphone in Real Ear technique (MIRE) calibration tables were used to determine a coupler to free-field correction factor to report free-field equivalent levels for individual frequencies from 0.25 to 6k Hz produced by the Verifit VF-1 (transfer function of the outer ear [TFOE] of the Verifit VF-1 measurements) [25]. 

Upon completion of audiological testing and Verifit probe tube measurements, the PLD user participants were asked to complete a listening habits survey that asked for information about use of personal listening devices. After measurements were finished, participants completed a second questionnaire querying their knowledge of potential risks of hearing loss. These questionnaires were adapted from Worthington *et al.* and were used to examine participants’ self-reported listening patterns as well as knowledge and education regarding noise exposure and hearing [8]. These questionnaires were completed following audiological testing to prevent education regarding noise exposure from impacting selected listening level. 

### 2.3. Health Related Fitness Testing

Specific markers of health related fitness included resting blood pressure and heart rate, blood lipids, body mass index (BMI), waist-to-hip ratio (W:H), hand grip strength, daily activity assessments via pedometer, and cardiovascular fitness using a sub-maximal VO_2_ test. All instrumentation was calibrated daily and tests performed by a trained technician. Blood pressure and heart rate were measured on the left arm of all subjects after they had been sitting for ten minutes using an Omron Intellisense Digital Blood Pressure Monitor, Model HEM-907xL (Omron, Hoffman Estates, IL, USA). Two readings were recorded, and averaged. If more than a 5% difference occurred between readings, the protocol called for a third reading to be taken after ten minutes. Blood lipid readings were collected using a Cholestech LDX analyzer, catalog number 02111239 (Cholestech Corporation, Hayward, CA, USA). Participants were instructed to fast for 12-hours prior to the blood lipid tests. Specific markers measured were total cholesterol (TC), high-density lipoproteins (HDL), low-density lipoproteins (LDL), triglycerides, non-HDL, TC/HDL and glucose.

Body composition was measured using a Tanida bioelectrical impedence body composition analyzer, Model TBF-300A (Tanida Corporation, Arlington Heights, IL, USA). Participants were instructed to remove their shoes and socks and step on the electrode plates on the scale portion. For all participants, weight of clothes was entered as 1.0 lb. and body type entered was “Athletic” for their gender. A SECA 210 cm stadiometer (Seca, Chino, CA, USA) was used to measure height. Specific markers recorded from the Tanida scale were weight, body fat percentage (FAT %), fat mass (FM) and fat free mass (FFM). Hip and waist measurements were taken using a Gulick II Tape, model 67020 (Northcoast Medical, Gilroy, CA, USA). Measures were taken around the umbilicus and widest area around the buttock. Handgrip strength was measured using a Takei physical fitness test grip strength dynamometer, model number 68812 (Niigata City, Niigata Prefecture, Japan). Participants were instructed to stand, hold the dynamometer at their side and to squeeze as hard as they could for approximately 2 sec. Right hand and left hand were measured twice and the better score was taken. Daily activity estimates were made by means of pedometer step data using the Yamax pedometer model SW-200 (Yamax Corporation, Tokyo, Japan). Participants were instructed to reset the pedometer each morning, wear the pedometer for all activities and record their daily steps at night for 14 days. The collection of pedometer steps was used to gain objective data for daily activity and also to validate self-reported exercise and daily activity routines.

Cardiovascular fitness was assessed by submaximal exercise tests whereby participants achieved a steady heart rate while walking for 3 min on a flat treadmill at a personally selected walking speed and 4 min at a five-degree incline. During this time, heart rate was measured with a Polar chest strap (T31, Polar, Lake Success, NY, USA) and a wristwatch. Minimum speed was no less than 4.3 mph and maximum speeds for men and women were 7.5 and 6.5 mph, respectively. According to the protocol, steady state heart rates were not to exceed 180 bpm for either gender. The protocol was modified slightly by having the participants ‘warm-up’ by walking at 3 mph for the first minute. Heart rate was measured with a Polar chest strap (T31) and wristwatch. At the completion of the test, the subject’s body weight in kilograms (BW), speed (mph), HR at termination (bpm) and gender (0 = Female, 1 = Male) were used to calculate an estimated VO_2_ max based on the well-known relation between heart rate and VO_2_ max. All subjects successfully completed the single-stage submaximum test and a reliability measure between VO_2_ max values predicted from this submaximal test and a true maximal test in a subsample (*n* = 20) was very strong (*r* = 0.86) [26]. Reliability was calculated using the equation:

Predicted VO_2_max = 54.07−0.1938 (BW) + 4.47 (speed in mph) – 0.1453 (HR on bpm) + 7.062 (gender: female = 0, male = 1) (1)

### 2.4. Statistics

Descriptive statistics were performed on the data set using SAS Software Version 9.2 for Windows (Statistical Analysis System, Cary, North Carolina, USA). The data were first summarized and examined for outliers and consistency. Several preparatory steps were taken to ready the data for analysis. Usage times were calculated by multiplying self-reported hours per day by days per week of PLD usage. Two samples of Verifit SPL values converted to A-weighted free field equivalents were each averaged for comparison to noise exposure standards and to pure tone threshold data. Pure tone thresholds in HL were analyzed in two ways as dependent variables: traditional frequency range (0.25–8 kHz) and high-frequency (HF) range (4–8 kHz).

## 3. Results and Discussion

### 3.1. Participant Information

A total of 119 participants, including 72 females and 43 males aged 18–84 participated in this study. Four participants were excluded for failing to complete all portions of audiologic and health- related fitness testing leaving a total of 115 participants, included in the analyses. The mean age of participants was 45 years with a SD of 16.5. Subjects were divided into two groups (Table 1) PLD users (*n* = 75) and non-PLD users (*n* = 40). The mean age of PLD non-users (52.78 years) was 12 years higher than the mean age of PLD users (40.71 years). For participants under age 50, the traditional frequency range mean was 10.97 dB HL, while the HF mean was 13.76 dB HL. Mean hearing thresholds between 0.25 and 8 kHz were poorer for participants over age 50, who had a mean of 14.58 dB HL and a HF-band mean of 22.81dB HL (Table 2). Twenty-three participants reported a history of ear infections, while 30 participants reported a family history of hearing loss. Thirty-one participants reported past or present tinnitus. Despite these reports, 100 of the participants presented with pure tone averages < 25 dB HL for octave frequencies from 0.25 to 8 kHz (within normal limits) and normal middle ear function determined by compliance and pressure measures. In the HF band means (4 kHz–8 kHz) an additional 12 participants were classified as having abnormal hearing (pure tone averages > 25 dB HL). 

**Table 1:** Participant demographics by gender and user-status.

PLD Status	N	# of Females	# of Males	Mean Age	Pure Tone Wideband Mean (dB HL)	Pure Tone HF-band Mean (dB HL)
PLD-user	72	42	33	40.71	11.98	14.91
Non-PLD user	43	30	10	52.78	15.23	23.39

**Table 2:** Traditional audiometric frequency means by age and frequency.

Age Group	Traditional Audiometry Mean (250K 500K 1K, 2K, 3K, 4K, 6K, 8K)	High-Frequency Band Means (4K, 6K, 8K)
< 50	10.97 dB HL	13.76 dB HL
50 +	14.59 dB HL	22.81 dB HL

### 3.2. Acoustic Variables

Due to the mean age mismatch of the PLD user group *versus* the non-PLD user group, age-adjusted ANOVA statistics were performed on the data to match the groups for age and rule out audiological changes that were the result of presbycusis rather than PLD user status or noise exposure. No differences were found between the PLD-user group and the non-PLD user group in the traditional frequency-band range (0.25–8 kHz,), the HF-band range (4–8 kHz) or DPOAEs. When examining the traditional frequency-band means, age was found to be statistically correlated (*p* < 0.001), while PLD listening status was insignificant (*p* = 0.7403). In the HF-band means, age was again the only significant variable contributing to hearing levels (*p* < 0.001), while PLD status was insignificant (*p* = 0.4787). A similar trend was observed when comparing the DPOAEs of PLD users and non-PLD users at both low and high intensity levels from 2 to 8 kHz. Age was a significant indicator of DPOAE levels at the low (*p* < 0.0001) and high (*p* < 0.0001) intensity levels, while PLD user status was deemed insignificant (*p* = 0.9479). 

The listening levels of the PLD user group were then examined in relation to hearing acuity. A total of 49 PLD users were included in this analysis. 36% of PLD users were excluded for failing for failing to complete all portions of audiologic testing. Of these 49 users, 94% were considered to have normal traditional frequency-band means (pure tone levels < 25 dB HL), while 6% were found to have abnormal levels (pure tone levels > 25 dB HL). In the HF range (4 kHz–8 kHz), 86% were found to have HF means in the normal range, while 14% of participants HF means were considered abnormal. Age (*p* < 0.0001) and listening level in silence (*p* = 0.0288) were the only two variables found statistically related to traditional frequency means. For the HF-band means, only age (*p* < 0.0001) was found to be significant. 

DPOAEs of the PLD user group were then examined. For the 27 participants who completed DPOAE testing, probe-microphone and PT testing, age was the only variable significant at the moderate (*p* = 0.0002) and high (*p* = 0.0007) intensity levels. Listening at harmful levels in silence had no significance on DPOAEs at the moderate 2–8 kHz (*p* = 0.2051) or loud 2–8 kHz (*p* = 0.0912) intensity levels. The second measurement in the presence of 75 dB SPL background noise revealed that age was again the only significant influence on the traditional frequency range means (*p* < 0.0001) as well as HF-band means (*p* < 0.0001). Participants who listened at levels above 80 dBA in noise showed no difference in hearing levels or DPOAE values at the moderate or high intensity levels. 

Finally, survey responses for the 51 PLD users and 45 PLD non-users were analyzed. These surveys examined variables contributing to hearing acuity such as age, audiology history, hearing protection use, and presence of tinnitus. Additional isolated questions targeted information specific to PLD users including exposure to noise and duration and frequency of listening to PLDs. Nineteen participants were excluded for failing to complete all parts of the survey. As seen in Table 3, select variables found to be a significant association for traditional frequency-band means for PLD users included age, years listening to PLD and hours listening to a device per week. Participants with increased age or those who listened to PLDs for more than 8 hours per week were found to have worse hearing (*p* = 0.0111). Contrary to our hypothesis, those who had used a PLD for a greater number of years were more likely to have better traditional frequency-band means when matched for age, than non-PLD users. However, for HF-band means of PLD users, variables found to significantly influence hearing levels included age, the use of hearing protection, and years listening to their device (Table 4).

**Table 3:** Variables contributing to wideband pure tone means for PLD users.

	Traditional Audiometric Frequency Means	
Parameter	*t* Value	Pr > |*t*|
Age	8.49	<0.0001
Total Number of Noise History Environments	−2.01	0.0502
Absence of Tinnitus	−1.70	0.0958
Family History of Hearing Loss	1.95	0.0572
Years Listening to Device	−2.31	0.0253
Hours Listening to Device/Week	2.65	0.0111

**Table 4:** Listening variables contributing to high-frequency band means for PLD users.

	High-Frequency Band Means (4 to 8k Hz)	
Parameter	*t* Value	Pr > |*t*|
Age	9.77	<0.0001
Total Number of Noise Environments	−2.14	0.0372
Years Listening to Device	−2.49	0.0164
Hours Listening to Device/Week	1.78	0.0822

For non-PLD users, similar analyses were done on survey questions. Age was the only contributing variable found to impact traditional frequency-band means. For HF-band means, significant influences included age (*p* < 0.0001) and consistent use of hearing protection. Participants who “always” used hearing protection were found to have better thresholds in the high frequency range (*p* = 0.02) while those who inconsistently wore ear protection showed no significant effect on hearing thresholds. 

### 3.3. Health-Related Fitness Variables

A total of 105 participants completed both the audiologic and health-related fitness portions of the study. Ten participants were excluded for failing to complete both portions of the study. For the traditional frequency-band means, age was found to be a significant contributing factor (*p* < 0.0001). Body Mass Index (BMI) was found to have a marginal positive correlation with traditional frequency-band means (*p* = 0.094). For the HF-band means, age (*p* < 0.0001) and W:H ratio (*p* = 0.04) were found to be contributing factors to hearing thresholds. W:H ratio was found to have a significant positive correlation with pure tone HF-band means (*p* = 0.004) after adjusting for age. 

### 3.4. Discussion

This study examined the dynamic relation between measures of hearing acuity, personal listening behaviors and biomarkers of cardiovascular health and fitness. Our results support the conclusion that age is the most significant factor contributing to hearing loss. Presbycusis, or age related hearing loss, is the most common sensory deficit in the elderly, and may occur as a result of various types of physiological degeneration in addition to the effects of noise exposure, medical disorder and genetics [27]. Presbycusis frequently begins as a high-frequency hearing loss and presents with clinical features of threshold shifts and decline of speech understanding [27]. This increase in high-frequency hearing loss as a result of aging is consistent with the finding that 12 participants showed traditional frequency-band mean hearing loss, while 24 showed hearing loss in HF-band means. Furthermore, participants over age 50 had traditional frequency-band means that were an average of 3.67 dB HL higher and HF-band means an average of 9.05 dB HL higher compared to their younger counterparts (Table 2). This supports the conclusion that older individuals have worse hearing, particularly in the high frequencies. 

As seen in Table 1, traditional frequency-band means for PLD users were better (11.98 dB HL) than the means for non-PLD users (15.23 dB HL). Despite these findings, no significant differences in hearing were found between the group of PLD users and the group of non-PLD users when analyzing the traditional frequency-band means, HF-band means and DPOAE values. These findings are consistent with reports from Berger *et al.* who reported that few people are at substantial risk of developing hearing loss from PLDs and instead, are at greater risk of developing hearing loss secondary to recreational noise exposure including hunting, power tools, operating motorized vehicles, attending sporting events and concerts and firing guns [11]. Contrary to Berger’s report, our data support an inverse correlation between exposure to leisure-noise and traditional frequency-band means (*p* = 0.05). In this case, individuals that exposed themselves to more different types of noisy environments tended to have better hearing. One hypothesis for this finding is that this study examined the relationship between total number of environments and hearing acuity, rather than examining individual environments and their relation to actual mean intensity levels of each exposure environment. Furthermore, frequency and duration of exposure in each environment reported in the survey was not co-factored into the analysis. Future research should examine the link between hearing acuity and individual noise environments, while considering the frequency, intensity and duration of the noise exposure within each context. 

Superior HF Pure Tone Thresholds (PTT) were observed for the five PLD users who protected themselves from noise damage through consistent use of hearing protection. No changes were observed for participants that “sometimes” or “rarely” wore hearing protection. The most common environments for the use of hearing protection included during construction, at concerts and at shooting ranges. Future research should continue to explore the possibility of DPOAE values as indicators of future NIHL. Nonetheless, no differences in mean DPOAE values at moderate and loud intensity levels were observed between those with frequent noise exposure, and those with limited noise exposure in the current study. This is contrary to the results of Seixas *et al.* who found that DPOAEs parallel deteriorations in PTTs and may worsen as a result of noise exposure [28]. 

When discussing the impact of PLD use on hearing, it is important to consider both the intensity and duration of listening. While NIOSH recommends a permissible exposure level of 85 dBA for 8 hours [29], the value of 80 dBA per-day was selected in the current study to parallel current international recommendations adopted by the World Health Organization for duration of noise exposure [30].

Consistent with the belief that intensity levels above 80 dBA are harmful to hearing, participants in the current study who listened at levels above 80 dBA in silence had worse hearing thresholds than those who listened at safe levels (Table 5). No differences were observed between those that listened at dangerous levels in the presence of noise and those that did not (Table 6). The mean intensity of listening level in silence and in noise was also considered according to gender. As seen in Table 5, participants’ volume level in silence was extremely variable. Although the majority of participants listened at “safe” levels, males were more likely to listen at louder and potentially dangerous levels in silence than females. In the presence of background noise (Table 6), nearly all groups increased their mean intensity volume. Furthermore, an additional 15 participants listened at levels deemed dangerous in the presence of background noise, including approximately equal amounts of males and females. 

**Table 5:** PLD listening levels in silence.

Silence Harmful	Gender	N	Wide band Mean(dB HL)	HF-band Mean(dB HL)	Mean Listening Intensity(dBA)	SD	Min Volume (dBA)	Max Volume (dBA)
No	Female	26	8.79	9.82	57.316	12.420	37.463	75.909
	Male	17	12.08	17.50	63.469	9.757	46.260	76.870
Yes	Female	2	18.57	27.50	90.130	9.381	83.497	96.763
	Male	6	14.76	17.22	82.984	2.334	80.097	85.800

**Table 6:** PLD listening levels in noise.

Noise Harmful	Gender	N	PT Wide band Mean(dB HL)	HF-band Mean(dB HL)	Mean Listening Intensity(dBA)	SD	Min Volume (dBA)	Max Volume (dBA)
No	Female	17	9.76	11.44	70.867	9.843	40.462	79.795
	Male	11	12.83	17.77	73.354	7.080	54.381	79.487
Yes	Female	11	9.07	10.52	87.149	4.640	81.371	95.070
	Male	12	12.73	17.11	87.675	6.630	81.556	101.856

Our findings indicate that PLD users who listen for greater than eight hours per week are at an increased risk for hearing loss compared with PLD users who listen less than eight hours per week. These results suggest that duration rather than intensity may increase subsequent risk of developing NIHL. This is consistent with the report of Meyer-Birsch who found that young adults who used their personal music system more than seven hours per week had poorer hearing thresholds compared with other young adults with less PLD use [9]. Contrary to our hypothesis, PLD users who had been listening to their devices for a greater number of years had better traditional frequency-band means and HF levels than PLD users who had been listening to their devices for less time, despite being adjusted for age. Different findings were reported by Peng *et al.* who found that 14% of young adults with long-term history of PLD use had hearing impairment. Of those participants with NIHL, incidence was highest in the subgroup that had been listening for five years, revealing that the risk of damage to hearing is increased as the length of listening time increases [31]. A possible explanation for this finding is that traditional frequency-band means were examined within the context of number of years listening alone. Future research should cofactor hours/week and intensity of listening along with years listening to obtain a more accurate representation of the relationship between PLD use and hearing health. 

Although researchers claim that high cardiovascular fitness positively influences the auditory system and in turn protects hearing sensitivity [32] the current study demonstrates that years of life are ultimately predictors of hearing health. In the traditional frequency-band means, a marginally significant correlation (*r* = 0.62) was found between hearing acuity and BMI (*p* = 0.09). BMI is an indicator of body fat composition taken from a person’s weight and height. It is calculated by dividing weight in pounds (lbs.) by height in inches (in) squared and multiplying by a conversion factor of 703. BMI values in the range of 18.5–24.9 are considered healthy while values above 25 are associated with being overweight or obese [33]. While BMI is commonly used as an indicator of obesity, it is also correlated with other measures of health including cardiovascular health and cholesterol. In this case, those with higher BMIs tend to have unfavorable lipid profiles including elevated triglycerides, and total cholesterol values [34]. Increased values of cholesterol and triglycerides in the blood increase the likelihood of blockage in blood vessels. This can result in excessive stress that can damage the heart in its effort to circulate blood and oxygen throughout the body, including two parts of the ear [35]. As seen in Table 7, mean BMI for participants in the current study was 25.21 with a SD of 4.62. This mean is considered to be on the border between “normal” and “overweight.” Nonetheless, Table 8 demonstrates that BMI for participants under age 50 was 24.39 (normal range) while mean BMI for individuals over age 50 was 26.31 (overweight). This indicates that in the current study, older individuals were more likely to have BMIs considered overweight than their younger counterparts. Furthermore, individuals who had lower (superior) hearing thresholds were also more likely to have lower BMI values. This is consistent with the finding that those with better overall health tend to have better hearing acuity. 

**Table 7:** Mean measures of health-related variables across participants.

Variable	Mean	SD	Minimum	Maximum
BMI (kg/m^2^)	25.21	4.62	18.46	38.92
Waist-to-hip ratio	0.84	0.09	0.60	1.09
VO_2_ Max (ml/kg/min)	34.54	8.76	14.80	63.90
Physical Activity (steps/day)	8714	3702	3185	18,257

**Table 8:** Mean measures of health related variables and hearing according to age.

Age	Variable	Mean	SD	Minimum	Maximum
50 +	BMI	26.30	5.10	18.7	38.1
	Waist-to-hip	0.87	0.08	0.7	1.1
	VO_2_ Max	30.90	5.40	17.6	42.7
	Pure Tone Mean 0.25–8 kHz	14.60	8.50	−10.0	75.0
	Pure Tone Mean 4–8 kHz	22.80	6.00	−10.0	75.0
≤50	BMI	24.39	4.06	18.5	38.9
	Waist-to-hip	0.8	0.08	0.6	1.1
	VO_2_ Max	37.2	9.80	14.8	63.9
	Pure tone Mean 0.25–8 kHz	11.00	2.40	−10.0	110.0
	Pure tone Mean 4–8 kHz	13.80	.86	−10.0	110.0

Using the HF-band means, an association was found between hearing acuity and W:H ratio. Normal W:H ratio measurements are: ≤0.80 for women and ≤0.90 for men [36]. As seen in Table 6, mean W:H ratios were 0.839 with a SD of 0.08. Nonetheless, mean W:H ratio was 0.875 for participants above age 50 while it was 0.813 for individuals below age 50. This signifies that younger individuals were more likely to have healthier W:H ratios than their older counterparts (Table 7). Furthermore, individuals who had lower (superior) hearing thresholds from 4 to 8 kHz also had smaller W:H ratios, indicating that they carried more of their weight on their hips (pear shaped) than around their waist (apple shaped). Research has shown that individuals who carry their weight on their hips rather than their waist have a lower risk of developing diabetes, cardiovascular disease (CVD) and other complications of metabolic syndrome. A study by Balkau explored the relationship between waist circumference (WC), CVD and diabetes mellitus (DM) in a sample of 168,000 patients ages 18–84 in 63 countries found that abdominal obesity, measured by WC, showed a graded relationship with both CVD and DM at all levels of BMI. In this case, both men and women with larger WC were more likely to develop CVD and DM than those with smaller waist circumferences [37]. 

These findings support the conclusion that improved health-related fitness as indicated by healthy body composition in particular, is associated with superior hearing in the H-F band. Exposure to excessive noise affects the H-F sounds first. Nonetheless, the impact of high intensity noise on hearing may be minimized through regular exercise. It is hypothesized that high levels of health-related fitness reduce susceptibility to TTS by increasing blood flow and oxygen delivery throughout the body, including the ear [17]. By improving the circulation of oxygenated blood, the cochlea is less susceptible to the onset of vasoconstriction brought on by loud noise [1]. Finally, despite the fact that VO_2_ max was lower in the group of participants over the age of 50 compared with <50 year-old group, age became a more potent variable than VO_2_ max. In a separate study, Alessio and Marron reported that individuals with VO_2 _max values greater than 31 mL/kg/min for women, and greater than 34 mL/kg/min for men, reduced their susceptibility to metabolic and cardiovascular disease and also had better hearing acuity regardless of age [33]. This suggests that despite the possible benefits of cardiovascular fitness on hearing acuity, non-modifiable behaviors discussed previously may make age-related hearing loss inescapable. Further study of health variables and hearing levels should examine the effects of sustained, life-long physical activity on hearing thresholds.

## 4. Conclusions

This study examined the relationship between hearing levels, listening habits of PLD users and cardiovascular health and fitness variables for adults aged 18 to 80. Age was found to be a significant predictor with traditional frequency-band thresholds, HF thresholds and DPOAEs. PLD user status did not impact hearing acuity. Nonetheless, PLD users who used their devices for longer than 8 hours/week were more likely to have worse hearing levels than PLD users who listened with limited duration. Furthermore, PLD users who listened at safe (<80 dBA) rather than dangerous (>80 dBA) intensities in silence had better traditional frequency-band threshold levels than those who listened at higher intensity volumes. Specific indices of health behavior appeared to influence hearing levels more than others. A healthier (e.g., lower) BMI was marginally associated with favorable levels of traditional frequency-band thresholds, while a healthier (smaller) W:H ratio was strongly correlated with improved hearing in the high frequencies. It is well known that healthy levels of physical activity and cardiovascular fitness are inversely associated with BMI. Therefore, it is possible that physical activity and cardiovascular fitness may have less of a direct effect on hearing, but rather, indirectly influence hearing via physical activity-induced changes in BMI as well as other factors (e.g., triglycerides) known to influence hearing. In conclusion, the risk of developing NIHL over time is mediated by several audiological and non-audiological behaviors. One non-audiological behavior is health related fitness that may influence hearing thresholds. Although PLD use alone is not a major predictor of hearing thresholds, intensity and duration of listening may be significant indicators of hearing acuity.
